# Transcriptome Profiling of microRNA by Next-Gen Deep Sequencing Reveals Known and Novel miRNA Species in the Lipid Fraction of Human Breast Milk

**DOI:** 10.1371/journal.pone.0050564

**Published:** 2013-02-13

**Authors:** Erika M. Munch, R. Alan Harris, Mahmoud Mohammad, Ashley L. Benham, Sasha M. Pejerrey, Lori Showalter, Min Hu, Cynthia D. Shope, Patricia D. Maningat, Preethi H. Gunaratne, Morey Haymond, Kjersti Aagaard

**Affiliations:** 1 Department of Obstetrics and Gynecology, Division of Maternal-Fetal Medicine, Baylor College of Medicine, Houston, Texas, United States of America; 2 Department of Molecular and Human Genetics, Baylor College of Medicine, Houston, Texas, United States of America; 3 Department of Pediatrics, Division of Pediatric Endocrinology and Metabolism, Baylor College of Medicine, Texas Children’s Hospital, The Children’s Nutrition Research Center, US Department of Agriculture, Agricultural Research Service, Houston, Texas, United States of America; 4 Department of Biology and Biochemistry, University of Houston, Houston, Texas, United States of America; 5 Department of Molecular and Cell Biology, Baylor College of Medicine, Houston, Texas, United States of America; 6 Department of Pathology, Baylor College of Medicine, Houston, Texas, United States of America; University of Melbourne, Australia

## Abstract

While breast milk has unique health advantages for infants, the mechanisms by which it regulates the physiology of newborns are incompletely understood. miRNAs have been described as functioning transcellularly, and have been previously isolated in cell-free and exosomal form from bodily liquids (serum, saliva, urine) and tissues, including mammary tissue. We hypothesized that breast milk in general, and milk fat globules in particular, contain significant numbers of known and limited novel miRNA species detectable with massively parallel sequencing. Extracted RNA from lactating mothers before and following short-term treatment with recombinant human growth hormone (rhGH) was smRNA-enriched. smRNA-Seq was performed to generate 124,110,646 36-nt reads. Of these, 31,102,927 (25%) exactly matched known human miRNAs; with relaxing of stringency, 74,716,151 (60%) matched known miRNAs including 308 of the 1018 (29%) mature miRNAs (miRBase 16.0). These miRNAs are predicted to target 9074 genes; the 10 most abundant of these predicted to target 2691 genes with enrichment for transcriptional regulation of metabolic and immune responses. We identified 21 putative novel miRNAs, of which 12 were confirmed in a large validation set that included cohorts of lactating women consuming enriched diets. Of particular interest, we observed that expression of several novel miRNAs were altered by the perturbed maternal diet, notably following a high-fat intake (*p*<0.05). Our findings suggest that known and novel miRNAs are enriched in breast milk fat globules, and expression of several novel miRNA species is regulated by maternal diet. Based on robust pathway mapping, our data supports the notion that these maternally secreted miRNAs (stable in the milk fat globules) play a regulatory role in the infant and account in part for the health benefits of breast milk. We further speculate that regulation of these miRNA by a high fat maternal diet enables modulation of fetal metabolism to accommodate significant dietary challenges.

## Introduction

Breast milk is the ideal source of nutrition for mammalian infants [1], [2]. It is widely recognized that human milk-fed infants receive significant health benefits compared to those fed substitute milk preparations [3]. These benefits include a decreased risk of a wide range of diseases, including otitis media, meningitis, respiratory infections, sudden infant death syndrome, diabetes, cancer, and obesity, as well as higher neurocognitive behavior testing and intelligence scores [3], [4]. Moreover, the benefits of breastfeeding extend to mothers as well as mother-infant pairs [4]–[6]. For years, infant formula companies have struggled to replicate this unparalleled balance of lipids, oligosaccharides, and proteins necessary for infant nutrition. However, formula-fed infants do not share the same health benefits as those partially or exclusively breast-fed [3]. The mechanisms underlying these beneficial effects are poorly characterized but may be the result of biologic macromolecules, including growth factors, antibodies, and small nucleic acids [7]–[9]. Each of these macromolecules is largely underappreciated and likely are not readily duplicated in substitute milk preparations, as they may be species specific.

Small, noncoding RNAs (smRNAs), including 20–24 nucleotide (nt) microRNAs (miRNAs), are critical, highly conserved mediators of posttranscriptional gene regulation and modulate gene expression in eukaryotes [10]. miRNAs have been described as functioning transcellularly with ubiquitous expression in human tissues and fluids and have been previously isolated in cell-free and exosomal form from bodily liquids (serum, saliva, urine) [11]–[16] and various bodily tissues including mammary tissue [17]–[18]. miRNAs play pivotal roles in regulating diverse developmental processes by targeting mRNA for translational repression, cleavage, or destabilization [19]. Recent studies have revealed that transcellularly acting miRNAs may play salient roles in the development of diabetes [20], and cancer [15]. Transcellular acting miRNAs are also a current focus of cardiovascular and cell-to-cell communication research [12]. miRNAs are crucial mediators of immune cognition [21], [22], and recent reports in immune models have demonstrated that in addition to their characteristic composition of proteins, T cell–derived exosomes contain RNA that can be stably transferred to the antigen-presenting cell, as an example of cell-to-cell genetic transfer, to modulate functional cognate immune interactions [11].

With these findings in mind, we speculated that the miRNAs in breast milk modulate infant physiology, immunity, and feeding behavior. Several studies have demonstrated that miRNA is present in bovine milk [23]–[25] and in whole human breast milk, including within milk fat globules [7], [9], [17], [26]. However, to our knowledge, no studies have interrogated for novel miRNAs employing Next-Gen deep sequencing discovery pipelines. Given that array analysis interrogates miRNAs that have been previously described in other research findings, and therefore are likely to be missing miRNA species limited to newly interrogated tissues or fluids, we sought in the present study to take an unbiased sequencing-based approach to identify novel species in RNA-enriched milk fat globules. We reasoned that since miRNAs bind a large number of targets, extensive mapping of both known and novel miRNA species to the 3′ untranslated region (UTR) of gene targets may in part explain the potential significance of miRNAs in breast milk.

In the current study, we hypothesized that a massively parallel sequencing-based approach would not only definitively identify miRNA in breast milk, but also expand the number of known miRNAs in human milk and concomitantly enable the identification of novel miRNAs. These miRNAs, both previously described and novel, could thereafter be mapped to target gene pathways to reveal potential molecular and physiologic functions which might account for the observed nutritive, cognitive, and immunity-based benefits that breast-fed infants are known to receive. We further reasoned that the potential functional significance of those novel miRNAs could be surmised using quantitative measures in well-described cohorts of lactating women subjected to significant dietary manipulations.

## Results

### Subject Cohorts

**Figure 1:**
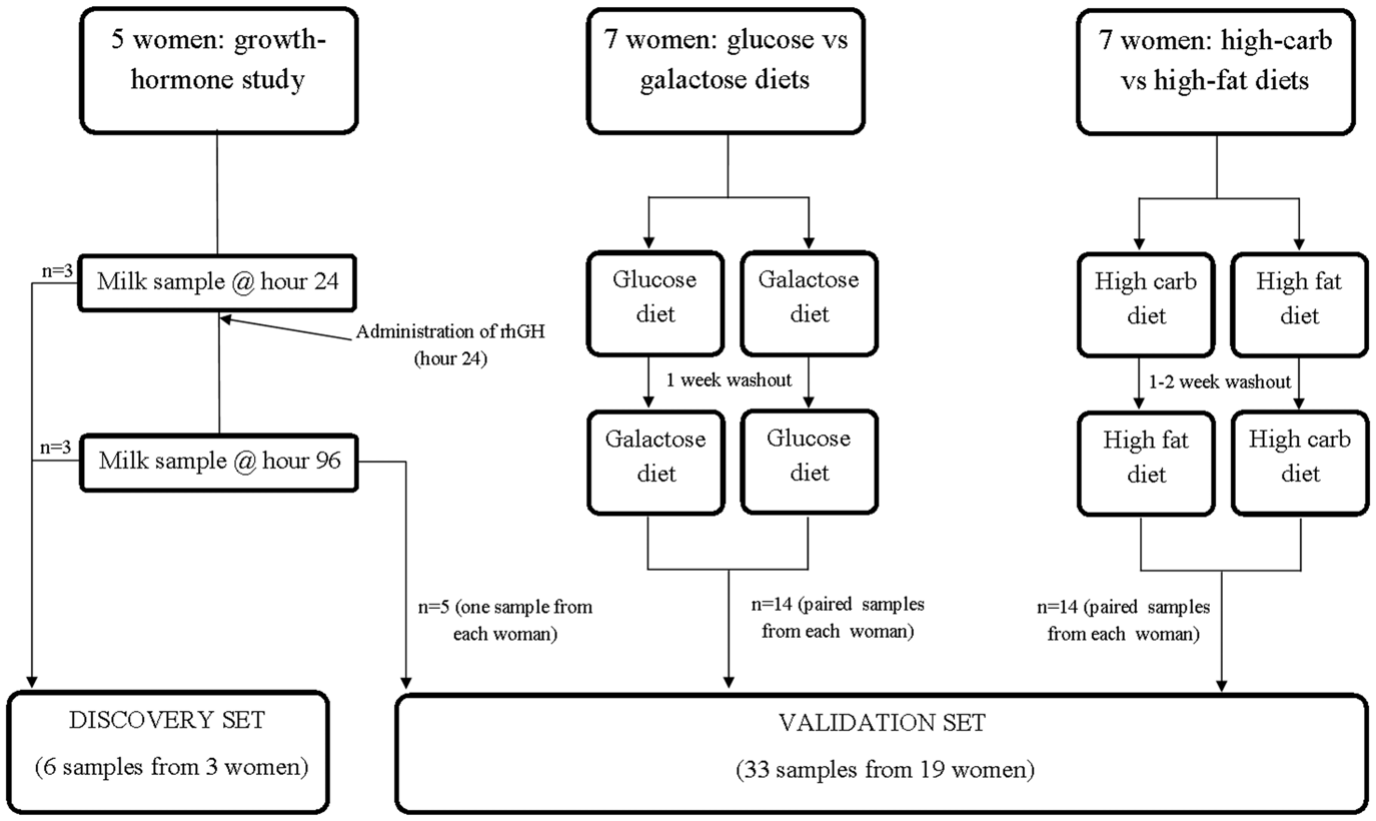
Flowchart outlining the acquisition of breast milk samples into sets for discovery, interrogation and validation. Each of these cohorts has been previously described and well-characterized [7], [27], [32]. Briefly, in the discovery set lean healthy women received weight-based recombinant human growth hormone for synchronization at 24 hours of study initiation for synchronization. The women continued to collect breast milk via pump every 3 hours for an additional 48 hours, and every 4 hours thereafter for a total of 96 hours studied. Breast milk samples used for the discovery set were collected at hour 24 and hour 96 (2 samples from each of 3 women, for a total of 6 samples). To both confirm and ascribe potential functional significance, two dietary manipulations were additionally employed in the validation set. These two cohorts were part of two crossover studies (whereby each subject serves as her own control following random sequence allocation) examining the effects of diet composition on milk quality, quantity, and carbohydrate expenditure. In the glucose-galactose cohort [27], 7 obese subjects consumed a diet of 15% protein, 50% carbohydrate, and 35% fat under controlled GCRC conditions. After an overnight fast, women were randomized to receive an isocaloric drink every 3 hours comprised of either glucose or galactose, the sugars in which provided them with 60% of their daily estimated energy requirement. In the high carbohydrate-high fat cohort [32] 7 lean subjects were randomly assigned to either a high-carbohydrate, low fat diet (60% carbohydrate, 25% fat, and 15% protein), or 2) a low-carbohydrate, high-fat diet (30% carbohydrate, 55% fat, and 15% protein), the caloric value of which was tailored to the woman’s required total energy intake (1.3 times basal metabolic rate). Breastfeeding occurred every 3 hours followed by milk expression via electric pump.

We studied three cohorts of lactating women (Fig. 1). Following IRB approval, a total of 5 lactating women were admitted to the General Clinical Research Center at Baylor College of Medicine as outlined in Methods, and baseline breast milk samples were collected every 3 hours for the first 24 hours. Since multiple hormones, including growth hormone, prolactin, insulin, and steroid and thyroid hormones, are necessary to ensure successful lactation, we opted to use a previously well-studied supraphysiological dose of rhGH (0.1 mg/kg/day) in short duration as a means to normalize across our cohort in an effort to facilitate comparison among subjects [26]. In the discovery set, 6 breast milk samples were obtained from 3 women (mean age, 23.3+/−3.2 years; mean BMI, 25.2+/−0.1) of self-identified Caucasian (*n* 1), Hispanic (*n* 1), or African-American (*n* 1) race. In the validation set, 33 breast milk samples were obtained from 19 women (mean age, 27.3+/−4.1 years; mean BMI, 27.7+/−1.39) of Caucasian (*n* 7), Hispanic (*n* 8), or African American (*n* 4) race or ethnicity. There was an expected statistically significant difference in participant’s weight between discovery (lean) and validation sets, as obese women were intentionally included by design of the original cohorts (notably, obese women studied in the glucose-galactose cohort) [27]. However, this difference did not manifest as a significant variation in BMI (Table 1).

**Table 1:** Characteristics of women in discovery and validation sets.

Characteristic	Discovery Set (*3 women*)	Validation Set (*19 women*)	*p* value
**Age (years)**	23.33±3.18	27.26±.090	0.34
**Weight (kg)**	**59.67±1.60**	**72.50±4.14**	**0.01**
**Height (cm)**	153.80±2.43	161.32±1.75	0.06
**BMI (kg/m^2^)**	25.22±0.13	27.74±1.39	0.09
**Weeks postpartum**	7.67±0.88	9.42±0.35	0.17

The discovery set was comprised of three women (1 Caucasian, 1 Hispanic/Mexican American, and 1 African American), rhGH synchronized at two time points, while the validation set arose from 19 lactating women (7 Caucasian, 8 Hispanic/Mexican American, and 4 African American). There were no statistically significant differences between the sets with respect to maternal age, height, BMI, or weeks postpartum. Weight was increased in the validation set by virtue of cohort design (p<0.01). However, this did not significantly confound composite BMI between the discovery and validation sets.

### miRNA Extraction

**Figure 2:**
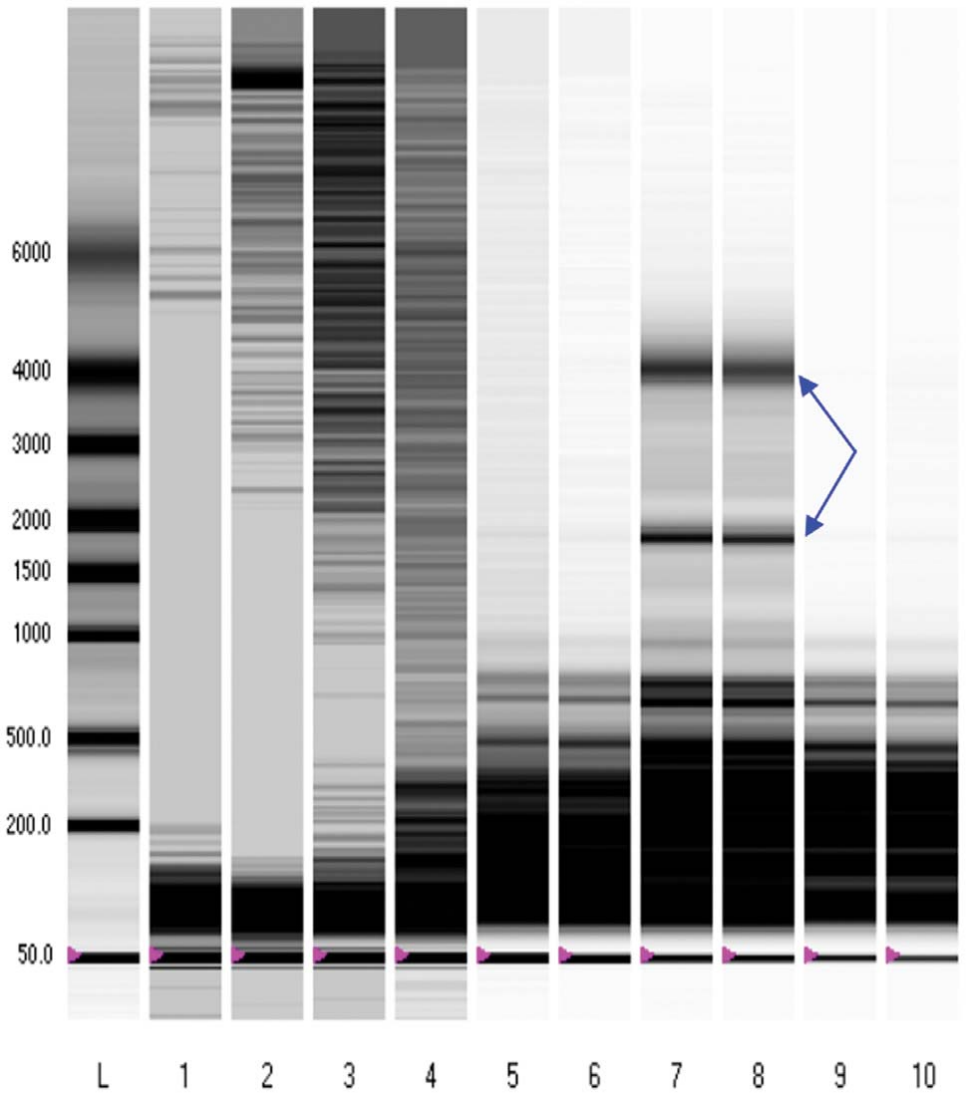
Electrophoresis of RNA extracted from breast milk samples. RNA integrity gels were employed in assessing for the integrity of RNA species. smRNA bands are shown, and ribosomal RNA identified with blue arrows. Lanes 1–4: Exosome precipitation with Exoquick system on whole fresh breast milk with a 2 hour precipitation (Lanes 1&2) versus precipitation overnight (Lanes 3 &4). Lanes 5&6: Trizol reagent smRNA preparation from lipid breast milk fraction. Lanes 7–10: mirVANA smRNA preparation from lipid fractions of fresh (Lanes 7 & 8) and previously frozen (Lanes 9&10) breast milk.

**Figure 3:**
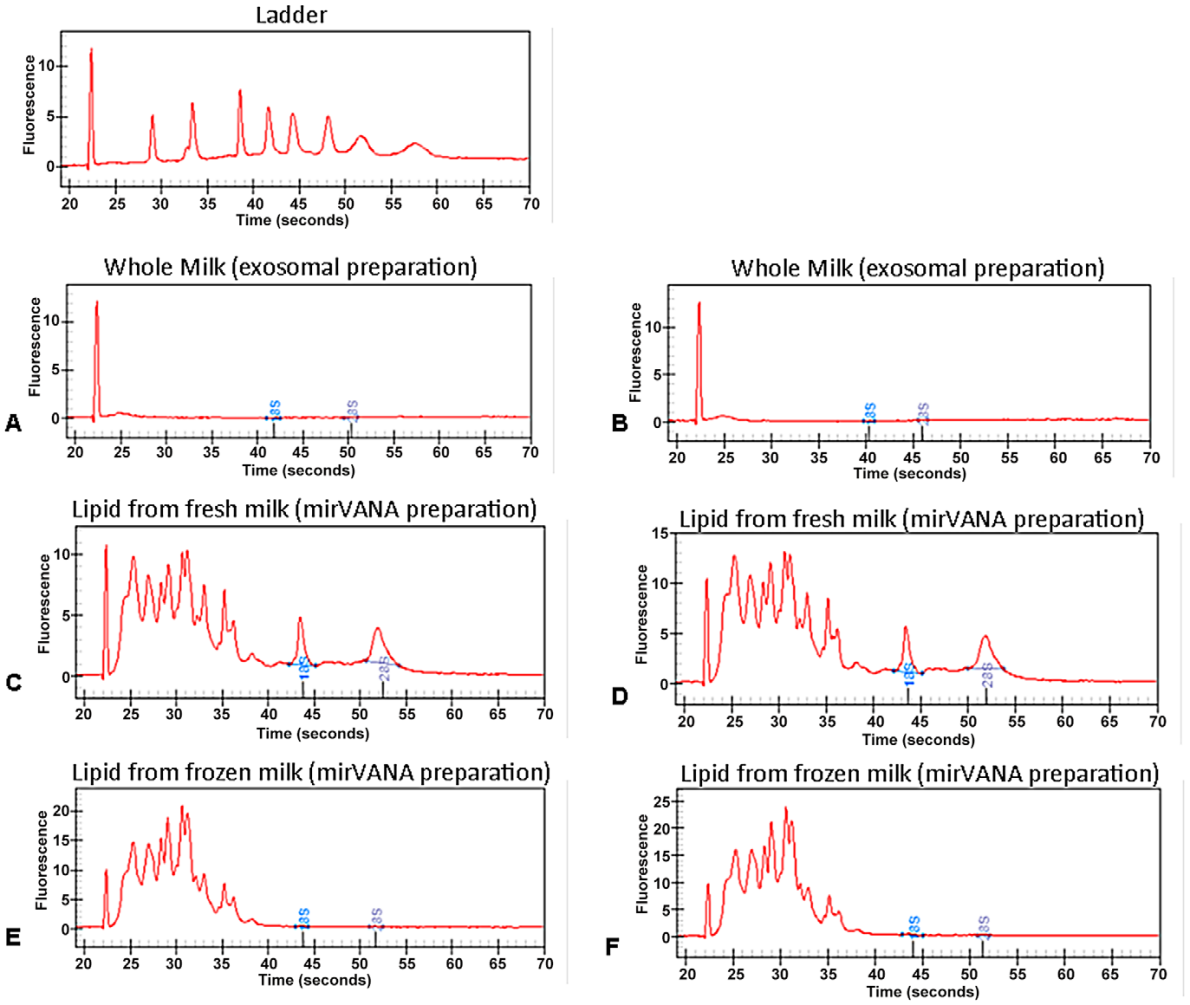
Chromatograms of breast milk preparations. Shown above are chromatograms from RNA demonstrated in gel electrophoresis from Fig. 2. (**A**) & (**B**): Whole breast milk (lanes 1 & 2), (**C**) & (**D**): Lipid fraction (milk fat globules) of fresh breast milk (lanes 7 & 8), (**E**) & (**F**): Lipid fraction frozen breast milk (lanes 9 & 10). These panels demonstrate enrichment for miRNA (blue boxes) in Trizol-treated, mirVANA isolated samples, which minimized the content of ribosomal RNA.

We first characterized optimal enrichment of yield and integrity of smRNA macromolecules from distinct fractions and preparations of human breast milk. Compared to the yield of smRNA from whole milk (Fig. 2, lanes 1 and 2), the lipid fraction of human milk containing milk fat globules was enriched for smRNA species (lanes 3 through 6). Moreover, the Trizol and mirVANA RNA isolation procedures gave the highest purity of miRNA with the lowest level of ribosomal RNA (Fig. 3). Based on these findings, the mirVANA isolation of frozen lipid fraction breast milk samples stabilized with Trizol were used for all subsequent experiments with both our discovery and validation sets.

### Identification of known miRNAs and their Targets

After small RNA sequencing with the Illumina GA-I Analyzer, a total of 124,110,646 usable reads were obtained from 6 breast milk samples (Supporting Information S1, Table S1). We processed these reads using our previously described pipeline [28]. Of these reads, 31,102,927 (25%) exactly matched to known miRNAs in miRBase 16.0, and 74,716,151 reads (60%) were matched using sequential relaxing (match to +4, and loose matches) (Supporting Information S1, Table S2). These matches corresponded to 308 of the known 1018 previously described miRNAs (Supporting Information S1, Table S3). The 10 most abundant of these previously described miRNAs are presented in Table 2.

**Table 2:** The 10 most abundant previously described (known) miRNAs in milk fat globules from human breast milk.

miRNA	Reads with exact match to known miRNA (miRBase 16.0)	Number of targets based on shared TargetScan and miRanda predictions
**hsa-mir-148a**	7,522,165	425
**hsa-let-7a**	3,500,919	529
**hsa-mir-200c**	2,946,303	664
**hsa-mir-146b-5p**	2,865,314	89
**hsa-let-7f**	2,845,728	531
**hsa-mir-30d**	2,076,325	890
**hsa-mir-103**	1,444,048	332
**hsa-let-7b**	1,012,530	527
**hsa-let-7g**	604,978	527
**hsa-mir-21**	493,693	166

The complete set of read counts with exact matches to known and previously characterized miRNAs are presented in Table S3. The number of targets predicted by TargetScan and miRanda for the 10 most abundant miRNAs are presented here. Gene symbols for targets predicted for all expressed miRNAs (Supporting Information S1, Table S4) and the top 10 expressed miRNAs (Supporting Information S1, Table S5) as well as enriched Gene Ontology (Supporting Information S1, Table S6, S8) and KEGG Pathways (Supporting Information S1, Table S7, S9) are presented in the supplemental materials.

To investigate the functional role of miRNA in human breast milk fat globules, we used TargetScan [29] and miRanda [30] predictions to identify miRNA gene targets. All miRNAs with an exact match to miRBase targeted 9074 genes predicted by both TargetScan and miRanda (Supporting Information S1, Table S4). The 10 most highly expressed miRNAs based on read counts targeted 2691 genes of interest that both algorithms predicted (Supporting Information S1, Table S5).

**Figure 4:**
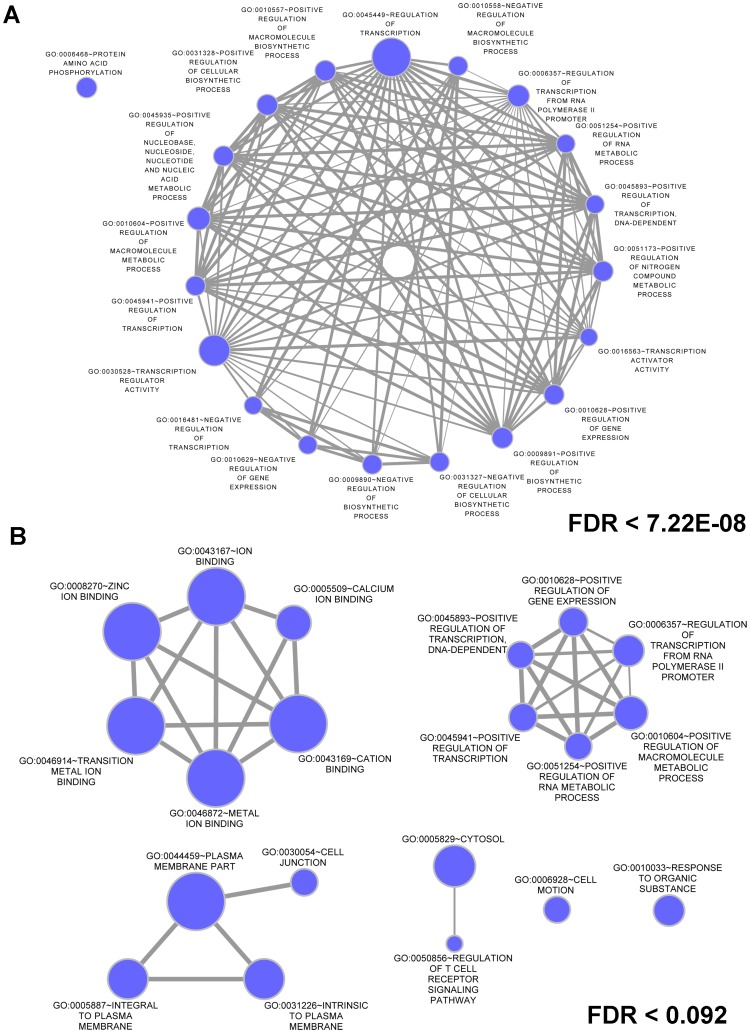
Graphs of top 20 enriched GO terms for miRNA targets. The top 20 enriched GO terms based on FDR (FDR levels shown) were graphed for gene targets of the top 10 expressed known miRNAs (A) or the validated novel miRNAs (B). GO terms related to the regulation of transcription, metabolic processes, and biosynthetic processes were highly represented among the known miRNA targets and form a connected graph consisting of 19 of the top 20 GO terms (A). A connected graph of terms related to regulation of transcription and metabolic processes was also present for targets of the novel miRNAs (B). For the novel miRNA targets, connected graphs of terms related to the plasma membrane, “regulation of T cell receptor signaling pathway”, and ion binding were also identified. The enrichment for “calcium ion binding” is of particular interest given the importance of calcium as a constituent of breast milk. Graphs were generated using the Cytoscape [47] Enrichment Map plugin [48].

For genes targeted by all miRNAs, enrichment analysis using the Database for Annotation, Visualization and Integrated Discovery (DAVID) [31] revealed 316 Gene Ontology (GO) terms (Supporting Information S1, Table S6) and 16 Kyoto Encyclopedia of Genes and Genomes (KEGG) pathways (Supporting Information S1, Table S7) enriched at a false discovery rate (FDR) <0.01. Similar analysis of the 10 most commonly expressed miRNAs showed enrichment for 145 GO terms (Supporting Information S1, Table S8) and 8 KEGG pathways (Supporting Information S1, Table S9). The top 20 enriched GO terms based on FDR for gene targets of the top 10 expressed miRNA were visualized as a graph with the node size representing the number of genes present in the GO term and the edge thickness representing the overlap between genes in different GO terms (Fig. 4A). GO terms related to the regulation of transcription, metabolic processes, and biosynthetic processes were highly represented among the enriched terms and form a connected graph consisting of 19 of the top 20 GO terms. This pattern of GO term enrichment was also seen for genes targeted by all miRNAs with “regulation of transcription from RNA polymerase II promoter” being the most significantly enriched term with 533 genes and 285 of these genes were involved in positive regulation. Other significantly enriched GO terms were involved in axon- or neuron-related terms. Notable KEGG pathways showing enrichment were “Insulin signaling pathway” and “Axon guidance”.

While no single immunity–related GO terms or KEGG pathways were significantly enriched at a FDR<0.01, DAVID clustering of GO terms or pathways showed a cluster of GO terms related to immune development and T and B cell function with an enrichment score of 2.17 (Annotation Cluster 52, Supporting Information S1, Table S10). Using the less stringent Benjamini multiple testing correction, we found that several of the terms in this cluster, including “immune system development,” were significant at <0.01. DAVID clustering of KEGG pathways showed a cluster of immune cell signaling pathways with an enrichment score of 1.75 (Annotation Cluster 3, Supporting Information S1, Table S11).

### Identification and Validation of Novel miRNAs in Human Breast Milk

After comparison to miRBase to identify known miRNAs and performing additional filtering, we found that 4759 reads corresponded to 21 potentially novel miRNAs (Table 3 and Supporting Information S1, Table S12, S13). TargetScan was used to predict gene targets for the 12 novel miRNAs that were validated (see *Validation* below). After filtering TargetScan predictions for those with a context+ score percentile of ≥90, 3554 gene targets were identified (Supporting Information S1, Table S14). GO enrichment showed 9 terms significantly enriched at FDR <0.01 (Supporting Information S1, Table S15), but KEGG Pathway enrichment analysis did not show any pathways enriched at the FDR <0.01 significance level (Supporting Information S1, Table S16). The top 20 GO terms associated with genes targeted by the novel miRNAs were visualized as a graph (Fig. 4b). Similar to the graph for known miRNA targets, a connected graph of terms related to regulation of transcription and metabolic processes was present. Connected graphs of terms related to the plasma membrane, “regulation of T cell receptor signaling pathway”, and ion binding were also identified. The enrichment for “calcium ion binding” is of particular interest given the importance of calcium as a constituent of breast milk. With the exception of “integral to plasma membrane” and “intrinsic to plasma membrane”, all GO terms showing enrichment for genes targeted by the novel miRNAs were shared with those enriched for genes targeted by all known miRNAs.

**Table 3:** 21 potential novel miRNA species in human milk fat globules were identified from rhGH synchronized lactating women in our discovery set (n = 6) by massive parallel sequencing.

Novel miRNA	Number of discovery set samples with putative novel miRNA(*n* 6 in total)	Number of reads
**Novel-miR-102**	**5**	**2201**
**Novel-miR-79**	**5**	**1639**
Novel-miR-85	4	205
**Novel-miR-114**	**4**	**117**
Novel-miR-54	4	34
**Novel-miR-37**	**4**	**20**
**Novel-miR-67**	**3**	**94**
**Novel-miR-27**	**3**	**82**
Novel-miR-109	3	63
Novel-miR-123	3	53
Novel-miR-44	3	36
**Novel-miR-111**	**3**	**28**
**Novel-miR-120**	**3**	**24**
**Novel-miR-118.2**	**2**	**54**
**Novel-miR-62**	**2**	**24**
Novel-miR-112	2	22
Novel-miR-113	2	16
Novel-miR-138	2	14
**Novel-miR-126**	**2**	**12**
Novel-miR-38	2	11
**Novel-miR-68**	**2**	**10**

12 of 21 (57%) species were thereafter confirmed in our robust validated set (n = 33) and are designated **in bold**.

### Normalization of Abundance with rhGH Cohorts

As a second means of identifying the potential functional significance of the putative novel miRNA species we discovered in human breast milk fat globules, we sought to interrogate the relative abundance of these miRNA species following maternal dietary manipulations in previously described and well-characterized obese and lean subject cohorts [27], [32]. Samples from the rhGH-treated cohort [7], [26] were used to normalize values and derive fold increase among each dietary manipulation. Specifically, read counts for all identified known miRNAs were quantile-normalized, and Pearson correlations were performed (Supporting Information S1, Table S17). Pearson correlations in pairwise comparisons of pre- and post-rhGH treatment sequencing data for the same subject ranged from 0.96 to 1.0, and correlations across the entirety of the cohort ranged from 0.95 to 1.0 for all known miRNAs identified (Supporting Information S1, Table S17). As a second means of determining appropriateness of normalization, the Linear Models for Microarray Data (Limma) R package [33] was also applied to the sequencing data after quantile normalization; no differential miRNA expression between the pre- and post- rhGH treatment sequencing data was identified at an FDR <0.01 (Supporting Information S1, Table S18). The lack of evidence of an effect of rhGH treatment on miRNA expression confirms the appropriate use of the rhGH cohort for our subsequent comparisons between dietary manipulation cohorts.

### A High-fat Diet Significantly Varies Abundance of Novel miRNA Species in Human Milk Fat Globules

**Figure 5:**
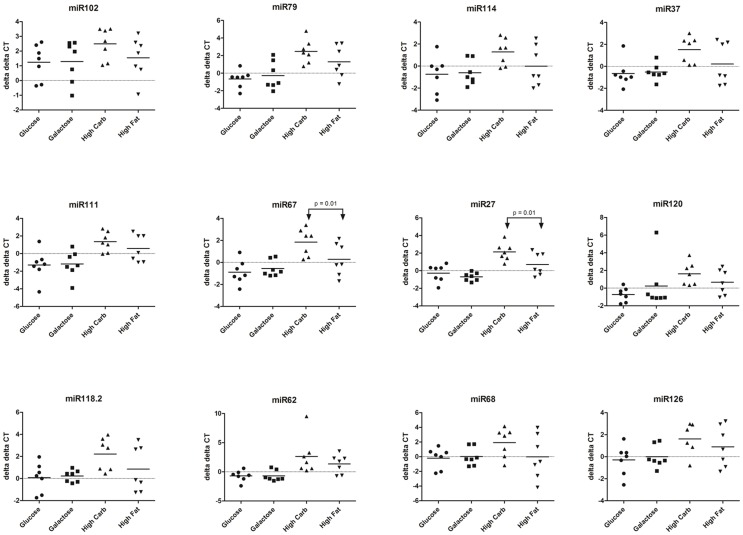
Delta delta CT comparing between rhGH (normalization) cohorts and dietary manipulated cohorts. When normalized to rhGH synchronized lean subjects, no significant differences were observed among obese lactating women on either high glucose or high galactose dietary manipulations. However, novel- miR-67 and novel-miR-27 species were observed to vary significantly (p = 0.01) following introduction of a maternal high fat diet in lean subject cohorts. This data is represented as fold change of cohorts with ANOVA for significance in Table S19.

After we determined an appropriate normalization set, we then sought to derive individual and cohort variation with respect to body habitus (obese versus lean) and dietary manipulation (relative glucose-galactose, and high fat-high carbohydrate). We observed significant variations (delta delta Ct following normalization to rhGH) only with putative novel miR-67 and miR-27 and only in women who were on a high-fat diet (Fig. 5). When considered as means among groups, we similarly failed to observe a significant effect of body habitus (obese versus lean), nor by dietary cohort except with high fat diet intake and only among putative novel-miR-67 and novel-miR-27 (ANOVA *p* = 0.01; Table S19). Novel-miR37 and miR-68 approached but did not reach significance (*p* = 0.07 and 0.08, respectively) (Fig. 5 and Supporting Information S1, Table S19).

### Putative Novel miRNA Species Abundance in Placental Tissue from Lean and Obese Gravidae

After we identified and validated novel miRNA in human breast milk, we next sought to determine whether these species are present in placenta. Like mammalian milk, the placenta is generated and only expressed during the interval of reproduction and has been found to play a pivotal role in modifying fetal growth and metabolism [34], [35]. Five of the novel miRNAs we identified were validated in both breast milk and placenta (novel-miR-62, −114, −118.2, −111, −120), and no miRNAs that did not validate in breast milk were validated in placenta (Supporting Information S1, Table S20). Although comparison in dietary cohorts could not be made, we found that the placental expression of detected novel miRNA in lean or obese subjects did not differ significantly (Fig. S1).

## Discussion

Researchers have understood for decades that human breast milk provides optimal postnatal nutrition for infants; however, the molecular mechanisms that enable the transfer of immunity- or metabolic- benefits previously described are incompletely understood. Given emerging evidence that transcellulary acting miRNAs are functionally present in serum, urine, and exosomes [12], [15], [20] and are crucial mediators of immune cognition [13], we sought to investigate both their presence and function in human breast milk. We found that not only are known and novel miRNAs present and stable in the milk fat globules, but that their measured expression is altered by a maternal high fat diet. Based on the findings of our target pathway analysis, we speculate that altered expression of miRNA species (from dietary manipulation) bears the potential to modify either mothers’ or infants’ metabolic pathways. This is consistent with the presumptive role of circulating miRNA biogenesis and secretion as pathophysiologic mediators, for which alterations in both concentration and composition of miRNA have been well correlated with cardiovascular morbidity and mortality [12], [15]. Zhou [36] recently speculated that miRNAs could be transferred through the gut in infants to aid in development of infants’ immune systems; we suggest that breast milk miRNAs could similarly be transferred through the infant gut and modulate gene expression, a mechanism explaining epigenetic maternal effects of offspring phenotype.

We found that the lipid fraction of breast milk, including milk fat globules, contained the highest quantity of miRNA and yielded the purest miRNA fractions. Previous study of mammalian genomes suggested that the most highly conserved proteins in milk were related to secretory processes and were contained within the milk fat globules [37]. Conversely, the most evolutionarily divergent proteins were associated with nutritional biogenesis. We found a significant number of conserved and known miRNA species that affect a large number of genes in a focused arena of pathways (Supporting Information S1 and Fig. 4) in the milk fat globules secreted from mammary cells, suggesting that the process of miRNA excretion in breast milk confers a potential evolutionary advantage.

Since it is not feasible to directly measure nor visualize a human infants’ physiologic responses along these miRNA-targeted pathways, we undertook a robust number of approaches aimed at elucidating and ascribing functional significance to both known and novel miRNAs we identified in breast milk. These included target gene pathway analysis of both known and novel miRNA species, analysis of specific and well-described perturbations in the maternal diet, and ascertainment of the presence of novel miRNA in another tissue unique to reproduction, the placenta.

With respect to the analysis of the mRNA transcripts that are targeted by these miRNAs, the 308 known miRNAs we found in breast milk target genes related to the regulation of gene transcription and metabolism as well as the establishment and refinement of immunity (Fig. 4A, Supporting Information S1, Tables S4 through S11). That the miRNAs in breast milk targeted these functional classes of genes suggests a molecular basis for the benefits experienced by milk–fed infants [3]–[6]. Additionally, many of the novel miRNAs discovered in our research target genes related to cancer. Target examples such as these suggest that miRNAs in breast milk could contribute to the decreased risk of cancer in humans that were milk-fed as infants [3]. Novel-miR-118.2 possibly targets odz, odd Oz/ten-m homolog 2 (Drosophila) (*ODZ2*); this family of proteins has been found in high quantities in central nervous system tissue, notably on brain axons, and is thought to be involved in cell-cell communication [38]. Based on these target predictions, it is interesting to further speculate that the presence of novel-miR-118.2 in breast milk may suggest a molecular mechanism for neurocognitive benefits derived from breast milk.

To more formally investigate the functional roles of these secretable miRNA species, we interrogated alterations in the relative composition of breast milk miRNAs in response to changes in the maternal diet. We used two previously described, well-characterized dietary manipulations in these experiments, in which breast milk samples from diet-varied women were normalized to rhGH-synchronized lean lactating women (Supporting Information S1, Tables S17 and S18). With respect to the appropriateness of our normalization cohort, Pearson correlations in pairwise comparisons between pre- and post- rhGH treatment sequencing data for the same subject ranged from 0.96 to 1.0, and correlations across the entirety of the cohort ranged from 0.95 to 1.0 for all known miRNAs identified. Moreover, Limma was applied to the sequencing data after quantile normalization and revealed no differential miRNA expression between the pre- and post- rhGH treatment sequencing data at an FDR <0.01 (Supporting Information S1, Table S18). These findings are consistent with Maningat’s earlier study of this cohort [*26*], which demonstrated that although the short-term administration of rhGH induced DNA synthesis, there was no significant difference in milk protein gene expression or milk production between pre- and post-treatment groups.

We have previously demonstrated that high-glucose diets in obese lactating women are associated with higher endogenous fat mobilization and oxidation during meal absorption compared to women fed high-galactose diets, whereas milk production, energy expenditure, and carbohydrate oxidation are no different [27]. This is in contrast to earlier findings pertaining to high fat relative to high carbohydrate consumption in lean lactating subjects, whereby a high-fat diet increases milk fat concentration as well as energy output and expenditure [32]. In this cohort, a high fat diet (and therefore a low carbohydrate diet, as the two diets were isonitrogenous and isocaloric) resulted in the lactating mothers adapting to low carbohydrate intake by decreasing carbohydrate oxidation. While we did not directly assess the infants’ physiology, our observations pertaining to miRNA excreted in the stable and transferable milk fat globules merit considerable attention.

Consistent with the overall effects of a high-glucose and –galactose diet on the physiology of the mothers and their milk, we did not observe significant variation in any of our novel miRNA species (Fig. 5 and Supporting Information S1, Table S19). However, the same is not true for women on a high-fat diet. Novel-miR-67 and -27 expression was significantly increased with high fat relative to high carbohydrate consumption (Supporting Information S1, Table S19) in the cohort as a whole, with as great as 6-fold effect sizes among individuals (Fig. 5). These findings suggest that breast milk has rich epigenetic potential in human infants. Alterations in the quantity and type of miRNA expression in breast milk could thus represent dynamic maternal regulation of infant gene expression based on changing environments and notably with significant maternal diet perturbations. Given that lactation helps mothers and offspring adapt to an ever-changing food supply, this would potentially provide an evolutionary advantage to the offspring [39]. Moreover, these findings are consistent with our prior observations in a non-human primate model on the impact of both the maternal and post-natal diet on modification in the offspring’s hepatic epigenome [40], [41]. In line with previous research showing that human milk constitution varies with gestational age at delivery as well as through the first six months of life [42], it is logical to speculate that miRNA expression would also change throughout the timeframe of lactation until weaning and future studies will interrogate miRNA profiles by virtue of gestational age at delivery.

Further insights into the potential biologic significance of our identified novel miRNAs can be gleaned by their predicted targeted transcripts. TargetScan was used to predict gene targets for the 12 novel miRNAs that were validated revealing 3554 gene targets for those with a context+ score percentile of ≥90 (Table S14). The top 20 GO terms associated with genes targeted by the novel miRNAs were visualized (Fig. 4B) and similar to known miRNA targets, a connected network of terms related to regulation of transcription and metabolic processes was observed. With the exception of “integral to plasma membrane” and “intrinsic to plasma membrane”, all GO terms showing enrichment for genes targeted by the novel miRNAs were shared with those enriched for genes targeted by all known miRNAs. Of noted interest, in addition to the transcription and metabolic regulatory network graphs of terms related to the plasma membrane, “regulation of T cell receptor signaling pathway”, and ion binding were uniquely prevalent among targets identified with mapping of our novel miRNA species. The enrichment for “calcium ion binding” is of particular interest given the importance of calcium as a constituent of breast milk [43], and further underscore the likely functional role these novel species heretofore not identified play in both infant and mother.

Finally, we measured the presence and abundance of the novel miRNAs we discovered in placental tissue from both lean and obese subjects to investigate the function of these miRNAs (Fig. S1 and Supporting Information S1, Table S20). Of our 21 putative miRNAs, none of the 9 species not validated in breast milk fat globules could be validated in placenta. However, of the 12 validated in breast milk, 5 of 12 novel miRNAs were validated in both breast milk and placenta (Novel-miR-62, −114, −118.2, −111, −120; Supporting Information S1, Table S20). These findings suggest that there may be common expression of miRNA among tissues and fluids which are uniquely produced in women during critical periods of reproduction and development, and may play a regulatory role in both the mother and her infant. This early line of investigation deserves significant attention in future studies.

Our study had several potential limitations. Given the high financial burden of sequencing, we elected to derive limited discovery sets and more robust validation sets. Thus, we performed sequencing on 6 samples from 3 patients in the discovery set, which likely does not completely represent the rich genetic diversity of the human species. However, given that each of our samples came from patients of different races, and that the validation sets were representative of the discovery set with both lean and obese subjects (Supporting Information S1, Tables S17 and S18), the limit to diversity was theoretically minimized. Another potential limitation of the present study was that potentially important miRNAs might reside in the whey portion of breast milk, which was not represented in our analysis of only the lipid fraction. However, our study is one of few that examines the RNA transcriptome of highly conserved milk fat globules [7], as many other studies of miRNA in breast milk have either discarded the milk fat globules in preparation for study of the whey fraction of milk [8], [36] or analyzed whole milk [9], [17], [25]. Our findings are thus an important contribution to the existing literature. It is possible that novel or known miRNA may have been degraded in the thaw process prior to purification; however, in previous work by Kosaka [9], the stability of miRNA has been demonstrated in at least 3 freeze-thaw cycles without changes in expression levels and thus we predict that the thaw effect was minimal to negligible. Although we could not biopsy normal infant tissue to more accurately define the functional significance of the novel miRNAs we discovered, their relative abundance in milk from women of various diets may lend merit to their presumptive significance. Finally, small errors in sequencing, though rare in this shotgun sequencing method, may have caused us to misidentify novel miRNAs or fail to identify additional miRNAs. Future studies should consider these limitations and examine the presence of these miRNAs in other breast milk samples from women of varying races and environments, as well as examine profile differences between whey and lipid fractions and fresh versus previously frozen milk. It is imperative that future studies deeply explore the dynamics of miRNA expression due to changing environments. However, despite these limitations, our study, novel in both construct and analysis, makes a pivotal leap in potentially explaining the molecular mechanisms for the benefits milk-fed infants receive.

Our findings indicate that massively parallel sequencing paired with robust analysis in well-described cohorts serves as a valid and powerful means of identifying known and novel miRNAs in human milk fat globules and characterizing their composition and abundance following diet alterations in lactating women. These miRNAs target critical pathways of the regulation of transcription, metabolism, and immunity. Thus, our findings elucidate a novel arena of molecular mechanisms that might explain the well-known benefits of human breast milk for human infants. We speculate that miRNAs function as a previously unrecognized means of dynamic maternal regulation of infant gene pathways and opens an exciting subfield of epigenetics that warrants deeper exploration.

## Methods

### Study Participants and Ethics Statement

All study protocols and consents were approved by the Institutional Review Board at Baylor College of Medicine (Houston TX). Females between 6 and 12 weeks postpartum of term (>37 weeks gestational age) with healthy infants gave written informed consent for breast milk donation after the nature and possible consequences of the study were explained. Exclusion criteria were age <13 years, preterm delivery, any chronic illness including diabetes, and any relatives with diabetes. Study participants were admitted to the General Clinical Research Center and collected breast milk at semi-hourly intervals while breastfeeding their own infants. Samples for both the discovery and validation sets were selected from 3 studies in which breast milk was obtained after women who were exposed to various research conditions (Fig. 1).

### Discovery Set [7], [26]

A total of 5 lactating women were admitted to the General Clinical Research Center at Baylor College of Medicine. At 0800 hours on the first day of the study, each woman breastfed her infant every 3 hours, and after 24 hours, received weight-based recombinant human growth hormone for synchronization. The women continued to collect breast milk via pump every 3 hours for an additional 48 hours, then every 6 hours thereafter for a total of 96 hours studied. Breast milk samples used for the discovery set were collected at hour 24 and at hour 96 (6 samples from 3 subjects).

### Validation Set

In addition to the 5 samples from the 5 women in the discovery set (obtained at hour 96), breast milk samples from two other cohorts of breastfeeding women were used for validation. These cohorts were part of two crossover studies examining the effects of diet composition on milk quality, quantity, and carbohydrate expenditure.

### Glucose vs. Galactose Enriched Diet Crossover Cohort [27]

7 lactating women (1 African American, 2 Caucasian, and 4 Hispanic) participated in the study. For the 3 days prior to admission to the General Clinical Research Center, the women consumed a diet of 15% protein, 50% carbohydrate, and 35% fat. Upon admission and after an overnight fast, the women were randomized to receive an isocaloric drink every 3 hours (starting at 0900 hrs) made of either glucose or galactose, the sugars in which provided them with 60% of their daily estimated energy requirement. Breastfeeding occurred every 3 hours followed by milk expression via electric pump. Breast milk samples studied in this investigation were collected 6 hours after the isocaloric drinks were administered (1500 hrs). As part of the crossover design, each woman participated in each study (glucose or galactose based drinks) with a 1 week washout period in between.

### High-carbohydrate vs. High-fat Crossover Cohort [32]

7 lactating women (2 African American, 2 Caucasian, and 3 Hispanic) were randomly assigned to either a high-carbohydrate, low fat diet (60% carbohydrate, 25% fat, and 15% protein), or a low-carbohydrate, high-fat diet (30% carbohydrate, 55% fat, and 15% protein), the caloric values of which were tailored to the woman’s required total energy intake (1.3×basal metabolic rate). Fructose content of both diets was constant at 20% of total carbohydrates. Each patient was provided complete meals/snacks in their home for the first 4 days of the study, thereafter they were moved to the General Clinical Research Center at Baylor College of Medicine, where they completed an additional 3 days of study consuming the same diets as was consumed during the previous 4 days. Breastfeeding occurred every 3 hours followed by milk expression via electric pump. After an overnight fast on day 3 in the hospital, small meals were provided every 15 minutes starting at 0900 hrs. Breast milk samples studied in our investigation were collected at 1600 hrs that day. As part of the crossover design, each woman underwent each 7 day diet (high carbohydrate or high fat) and milk collection with a 1 to 2 week washout period in between.

### Milk Collection and Storage

After infant feeding, breast milk was obtained from both breasts by a standard breast pump until the breasts were emptied. These samples were then stored on ice until centrifugation in RNAse free tubes at 4 degrees Celsius (3000 rpm for 10 minutes); thereafter the fat layer was transferred to a new RNAse free tube. TRIzol (Invitrogen Life Technology) was added to the samples for RNA stabilization, and the samples were stored at -80 degrees Celsius until RNA isolation.

### Initial RNA Extraction Techniques from Human Milk

For experiments determining the optimal enrichment and yield of smRNA from human milk, 3 different preparations methods were compared. For all 3 preparations, human milk was collected with standard breast pump and centrifuged at 2000 g for 10 minutes to remove large cellular debris. Preparations were analyzed using the Experion Standard-Sensitivity RNA Analysis kit (#700-7103, Bio-Rad USA); all run samples passed small RNA integrity parameters and were analyzed by gel electrophoresis.

1. ExoQuick™ Exosome precipitation with whole milk (System Biosciences Inc): precipitation solution was added to whole milk, and after either 2 hours incubation or overnight incubation, the solutions were centrifuged at 1500×g for 30 minutes, then 1500×g for 5 minutes. After discarding the supernatant, the pellet was then suspended in nuclease-free water and stored at −80°C until use.
2. Tri-Reagent RNA isolation with the lipid fraction of milk: the lipid fraction of whole breast milk was isolated and homogenized in Tri-Reagent solution, then extracted, precipitated, washed, and eluted per instruction from the manufacturer protocol (AM9738, Applied Biosystems).
3. mirVANA isolation of fresh and frozen lipid fraction: the lipid fraction of whole breast milk, both fresh milk and previously frozen breast milk, was isolated and homogenized in lysis solution extracted, precipitated, washed and eluted as described in the manufacturer protocol. (AM1560, Applied Biosystems).

### miRNA Extraction, Sequencing, and Identification

The Applied Biosystems mirVANA protocol (AM1560) was used for breast milk samples from the discovery and validation sets. RNA libraries were created from the mirVANA isolated samples and sequenced using the Illumina 1G Genome Analyzer to generate 36 nt sequencing reads. These reads were used to identify miRNAs using our previously described pipeline [28]. miRNAs were mapped against the public database miRBase release 16 for identification of known miRNAs. Reads that did not map to known miRNAs were mapped to the human genome (NCBI 36/UCSC hg18). Reads mapping exactly to the genome along with 100 bp flanking sequence were folded as RNA using the Vienna package [44] to identify hairpins. Reads within hairpins were further filtered to remove miRBase release 18 miRNAs, snoRNAs, scaRNAs, repeats as identified by RepeatMasker [45], and reads with >90% GC content [46]. Remaining reads that were present in at least two of the sequencing runs were identified as putative novel miRNAs.

### miRNA Target Identification

Gene targets for known miRNAs were identified using TargetScan [29] context+ scores and contributions for all conserved miRNA sites (http://www.targetscan.org/vert_61/vert_61_data_download/Conserved_Site_Context_Scores.txt.zip) and miRanda [30] good mirSVR score, conserved miRNA (http://cbio.mskcc.org/microrna_data/human_predictions_S_C_aug2010.txt.gz) target predictions. Targets predicted by both algorithms were identified to compile a stringent list of targets. This was done for both the top 10 most highly expressed miRNAs based on read count as well as all miRNAs that were identified in the sequencing data. The TargetScan tool was run on the putative novel miRNA sequences to identify targets for novel miRNAs.

### Gene Ontology and Pathway Enrichment Analysis

Gene Ontology (GO) and KEGG pathway enrichment were performed using DAVID software [31]. Graphs of the top 20 enriched GO terms were generated using the Cytoscape [47] Enrichment Map plugin [48].

### mirVANA miRNA Extraction from Placental Tissue

Placental tissue, previously acquired at time of delivery of lean and obese gravid women and immediately stored at -80 degrees Celsius, was homogenized in lysis buffer on ice prior to extraction, precipitation, and elution of miRNA as described in the manufacturer protocol (AM1560, Applied Biosystems), as to avoid release of RNAses from cellular compartments. Lean and obese subjects were defined by their pregravid body mass index (BMI), with a BMI >30 kg/m^2^ denoting obesity, and <25 kg/m^2^ denoting lean.

### Validation

QT-PCR was used to validate miRNA sequences of interest in both breast milk and placental. For the breast milk, samples obtained from the discovery set (growth-hormone synchronized women) and validation set (women in the two crossover cohorts plus women in the growth-hormone synchronized cohort) were purified as noted for sequencing; placental samples as noted above. Primers and probes were designed with the custom TaqMan® small RNA assays kit (Applied Biosystems, Carlsbad, CA) for the detection of miRNAs. TaqMan® putative mature miRNA (pmm) probes were designed as follows (all 5′ to 3′): Novel-miR-102 TCCATATCCCAACCTGTCAGAGTCT; Novel-miR-79TTTTTTGCTGGAACATTTCTGG; Novel-miR-54TAAGTTATCAAGGCATGAGAGA; Novel-miR-114GTGCGTGGTGGCTCGAGGCGGG; Novel-miR-37TTGGAGAGTGGGCAGCAGAGA; Novel-miR-111TGGGCGAGGGCGGCTGAGCGGCT; Novel-miR-67TTGAGGGGAGAATGAGGTGGAGA; Novel-miR-109ACGCGATTTGTAGCACAGACA; Novel-miR-27TCTCACCTGGCATAAGCAATT; Novel-miR-120TCTAGCAAGGGGCAGCTGCAGA; Novel-miR-123AGCAAAGCAAAGCTCAGTTGGA; Novel-miR-44TCAGCTACTACCTCTATTAGGA; Novel-miR-112TGAGAAAATGTAGAACCAAT; Novel-miR-113CAGGGCCTGGAACTCCAATGGGA; Novel-miR-118.2TTGAACTCGAGTTGGAAGAGGCG; Novel-miR-135ATTAGGTAGTGGCAGTGGAA; Novel-miR-138TCTGCACTCTATACGACCCAG; Novel-miR-62TGAGAGGGAGAAGGGGCTGCAG; Novel-miR-68CTGGTGAGCCCTGTGCTGTTCCAGGA; Novel-miR-26GCGATTGTAGATGTTATTGAT; Novel-miR-126GGGACCCAGGACAGGAGAAG. Values were normalized by Livak and Schmittgen 2^−ΔΔCT^ Method employing rhGH subjects for normalization and U6 snRNA (5′ GTGCTCGCTTCGGCAGCACATATACTAAAATTGGAACGATACAGAGAAGATTAGCATGGCCCCTGCGCAAGGATGACACGCAAATTCGTGAAGCGTTCCATATTTT3′) as an internal control. All data and statistical analysis was performed using Excel (Microsoft) and ANOVA (Partek). A p-value of <0.05 was considered statistically significant.

## Supporting Information

Figure S1 **Non-significant fold change of variation in placental expression among lean and obese cohorts of validated novel-miRNA species.** Graph of the placental expression fold changes for three miRNAs that were validated in both human placenta and breast milk. The miRNAs were extracted from placentas of both lean and obese patients. None of the p-values between the lean and obese groups were significant (novel-miR-62 p = 0.567076, novel-miR-114 p = 0.719292, novel-miR-118.2 p = 0.750468).
(DOC)

Supporting Information S1 Table S1. Summary of Sequencing Statistics. Table S2. Sequencing Statistics mapped to miRBase release 16. Table S3. Mapping frequencies for miRNA sequenced. Table S4. Gene targets for all expressed miRNAs with exact matches. Table S5. Gene targets for the top 10 expressed miRNAs with exact matches. Table S6. Gene Ontology classifications for genes targeted by all miRNAs. Table S7. KEGG pathway classifications for genes targeted by all miRNAs. Table S8. Gene Ontology classifications for genes targeted by the top 10 expressed miRNAs. Table S9. KEGG pathway classifications for genes targeted by the top 10 expressed miRNAs. Table S10. Gene Ontology term clustering as determined by DAVID for genes targeted by top 10 expressed miRNAs. Table S11. KEGG Pathway clustering as determined by DAVID for genes targeted by top 10 expressed miRNAs. Table S12. Putative Novel miRNAs. Table S13. Putative Novel miRNA hairpins (figures). Table S14. Validated novel miRNA TargetScan predictions. Table S15. Gene Ontology classifications for genes targeted by validated novel miRNAs. Table S16. KEGG Pathway classifications for genes targeted by validated novel miRNAs. Table S17. Pearson correlations between quantile normalized read counts from each sequencing sample. Table S18. LIMMA analysis for miRNA differential expression pre- and post rhGH treatment. Table S19. Fold change of putative novel miRNA species by virtue of maternal diet. Table S20. 21 novel miRNAs whose expression was measured in both human breast milk and placenta.
(XLS)
