# Supplementary material for: Transcriptome Profiling of microRNA by Next-Gen Deep Sequencing Reveals Known and Novel miRNA Species in the Lipid Fraction of Human Breast Milk
Source: PLoS One. 2013 Feb 13;8(2):e50564. doi: 10.1371/journal.pone.0050564 (PMC3572105; doi:10.1371/journal.pone.0050564)
Supplement: Figure S1 — Non-significant fold change of variation in placental expression among lean and obese cohorts of validated novel-miRNA species. Graph of the placental expression fold changes for three miRNAs that were validated in both human placenta and breast milk. The miRNAs were extracted from placentas of both lean and obese patients. None of the p-values between the lean and obese groups were significant (novel-miR-62 p = 0.567076, novel-miR-114 p = 0.719292, novel-miR-118.2 p = 0.750468). (DOC) [file pone.0050564.s001.doc]

***
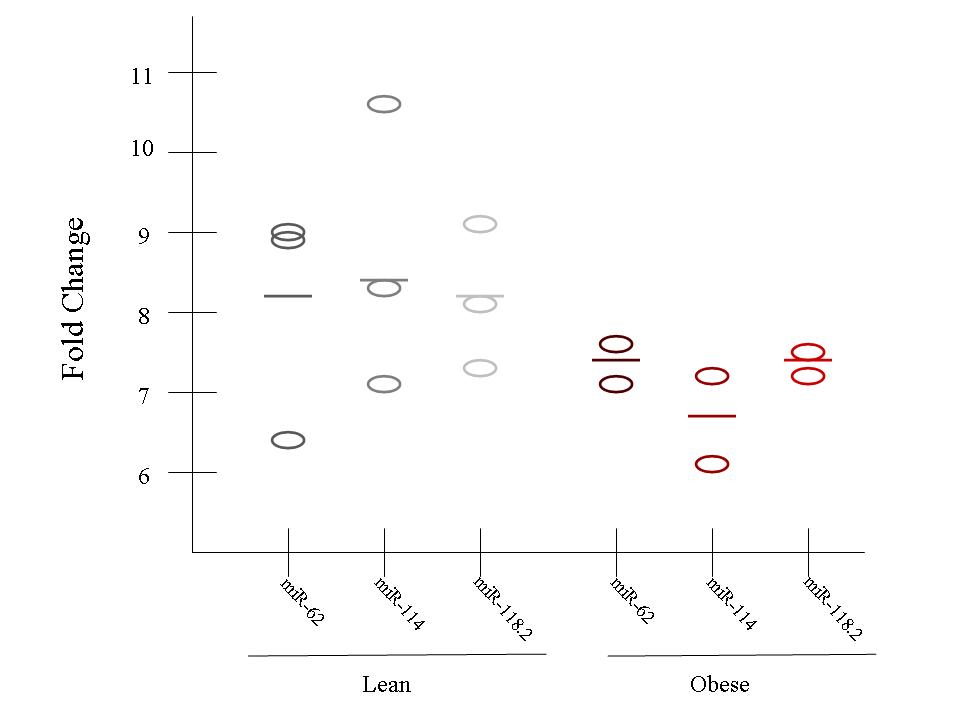
***

**Supplementary Fig S1. Fold change of variation in placental expression among lean and obese cohorts of validated novel-miRNA species.** Graph of the placental expression fold changes (Livak and Schmittgen 2^-∆∆CT^) for three miRNAs that were validated in both human placenta and breast milk. The miRNAs were extracted from placentas of both lean and obese patients. None of the p-values between the lean and obese groups were significant (novel-miR-62 p=0.567076, novel-miR-114 p=0.719292, novel-miR-118.2 p=0.750468).
